# Dual inhibition of anti-apoptotic proteins BCL-XL and MCL-1 enhances cytotoxicity of Nasopharyngeal carcinoma cells

**DOI:** 10.1007/s12672-022-00470-9

**Published:** 2022-02-03

**Authors:** Siti Fairus Abdul Rahman, Azali Azlan, Kwok-Wai Lo, Ghows Azzam, Nethia Mohana-Kumaran

**Affiliations:** 1 School of Biological Sciences, Universiti Sains Malaysia, 11800 Penang, Malaysia; 2 Department of Anatomical and Cellular Pathology and State Key Laboratory in Oncology in South China, The Chinese University of Hong Kong, Central Ave, Hong Kong; 3 Malaysia Genome and Vaccine Institute, 43000 Selangor, Malaysia

**Keywords:** Nasopharyngeal carcinoma, BCL-XL, MCL-1, BFL-1, BH3 mimetics, Spheroids

## Abstract

One of the many strategies that cancer cells evade death is through up-regulation of the BCL-2 anti-apoptotic proteins. Hence, these proteins have become attractive therapeutic targets. Given that different cell populations rely on different anti-apoptotic proteins for survival, it is crucial to determine which proteins are important for Nasopharyngeal carcinoma (NPC) cell survival. Here we determined the survival requirements for the NPC cells using a combination of the CRISPR/Cas9 technique and selective BH3-mimetics. A human apoptosis RT^2^ Profiler PCR Array was first employed to profile the anti-apoptotic gene expressions in NPC cell lines HK-1 and C666-1. The HK-1 cells expressed all the anti-apoptotic genes (*MCL-1, BFL-1, BCL-2, BCL-XL, and BCL-w*). Similarly, the C666-1 cells expressed all the anti-apoptotic genes except *BFL-1* (undetectable level). Notably, both cell lines highly expressed *MCL-1*. Deletion of *MCL-1* sensitized the NPC cells to BCL-XL selective inhibitor A-1331852, suggesting that MCL-1 and BCL-XL may be important for NPC cell survival. Co-inhibition of MCL-1 and BCL-2 with MCL-1 selective inhibitor S63845 and BCL-2 selective inhibitor ABT-199 inhibited NPC cell proliferation but the effect on cell viability was more profound with co-inhibition of MCL-1 and BCL-XL with S63845 and A-1331852, implying that MCL-1 and BCL-XL are crucial for NPC cell survival. Furthermore, co-inhibition of MCL-1 and BCL-XL inhibited the growth and invasion of NPC spheroids. Deletion of *BFL-1* sensitized NPC cells to A-1331852 suggesting that BFL-1 may play a role in NPC cell survival. Taken together co-inhibition of BCL-XL and MCL-1/BFL-1 could be potential treatment strategies for NPC.

**Supplementary Information:**

The online version contains supplementary material available at 10.1007/s12672-022-00470-9.

## Introduction

NPC is rare cancer worldwide but endemic in Asia especially in South-East Asia [1]. Concurrent chemoradiotherapy (CCRT) plus adjuvant chemotherapy has been the standard of care treatment for NPC since it was established by the intergroup 0099 [2]. However, treating patients with metastatic NPC is often a challenge as patients tend to develop resistance to cisplatin and radiotherapy. Hence, improved treatment strategies are needed for better patient survival.

The BCL-2 family of proteins is a critical regulator of the apoptosis pathway. The members of the family are grouped into pro-and anti-apoptotic proteins [3]. The anti-apoptotic proteins (BCL-2, BCLXL, BCL-w, MCL-1, and BFL-1) are up-regulated in many cancers and hence have emerged as potential therapeutic targets. Initial studies on the expressions of the BCL-2 family proteins in NPC focused on BCL-2. The immunohistochemistry (IHC) technique was unanimously employed to determine the expression level of BCL-2 in NPC tissues. Using IHC, BCL-2 expression was detected in 80% NPC tissues and 71% adjacent dysplastic lesions compared to only 37% in nasopharynx epithelial (NPE) and 30% in NPE of chronically inflamed nasopharynx patients [4]. In a separate study 31 of 51 NPC tumour samples tested, expressed BCL-2 [5]. High expression of BCL-2 was also observed in a large pool of NPC tissues of 148 samples compared to 164 samples of non-cancerous NPC tissues [6]. Patients with negative BCL-2 expression were reported to display better disease-free 5-year survival compared to patients with BCL-2 positive tumours [7].

Several studies attempted to target the anti-apoptotic proteins for NPC treatment but the study findings were unsatisfactory [8–10]. The problem lied largely on the failure to determine which anti-apoptotic proteins did NPC cells relied on for survival. A recent finding demonstrated that co-inhibition of MCL-1 and BCL-2 killed NPC cells in both in vitro and in vivo models [11]. However, the role of BCL-XL for NPC cell survival was not interrogated in the same study. Our study unveiled that co-inhibition of BCL-2 and BCL-XL did not induce cell killing in NPC [10]. This led to the re-evaluation of the utility of BCL-2 and BCL-XL as therapeutic targets in NPC. Study published by Xiang et al., raised two questions (1) do NPC cells depend on other anti-apoptotic proteins for survival? (e.g. MCL-1 or BFL-1); OR (2) do they depend on a combination of two anti-apoptotic proteins that are not partly targeted, by ABT-263, for example, MCL-1 and BCL-XL? This study primarily focused on investigating the functional relevance of MCL-1 and BFL-1, given the limited efforts in addressing their roles in NPC survival.

There are a few strategies to determine the dependencies of cancer cells to anti-apoptotic protein(s) namely the dynamic BH3 profiling technique [12], the CRISPR/Cas9 genome editing technique [13], and the chemical parsing approach using BH3-mimetics, given their selectivity in inhibiting specific anti-apoptotic proteins [14]. This study utilized a combination of the CRISPR/Cas9 technique and BH3-mimetics to delineate the contributions of the anti-apoptotic proteins for NPC cell survival. The functional relevance of *MCL-1* and *BFL-1* for NPC cell survival was first determined using the CRISPR/Cas9 technique and later the parental and *MCL-1*/*BFL-1* manipulated NPC cells were subjected to treatment with selective BH3-mimetics to complement the gene-editing studies and to also investigate the translational relevance of the BH3-mimetics for NPC management.

## Material and methods

### Cell culture

The HK-1 and C666-1 cell lines were cultured in RPMI 1640 medium (supplemented with 10% heated foetal bovine serum (FBS). Additionally, 10% (v/v) Glutamax was added to the RPMI complete medium that was used to grow the C666-1 cells. NPC cell lines HK-1 and C666-1 were authenticated using the AmpFISTR profiling [15]. All cell culture consumables were purchased from Thermo Fisher Scientific, MA, USA.

### Custom RT^2^ profiler PCR array

Total RNA from the HK-1 and C666-1 parental cell lines were extracted with TRIzol reagent (Invitrogen). RNA purity was quantified using the NanoDrop spectrophotometer (Thermo Fischer Scientific) and the PCR array (customized human apoptosis profiler array designed by Qiagen, Hilden, Germany) was conducted as per the manufacturer’s protocol. Data were analysed using web-based analysis tools.

### sgRNA design and cloning

The CHOP CHOP design software was employed to design sgRNAs (https://chopchop.cbu.uib.no/)*.* Two sgRNAs were designed for each gene of interest. The sgRNAs were cloned into expression vector plasmid, pSpCas9(BB)-2A-Puro (Px459) (Addgene). The sgRNAs oligo design included a 4-bp overhang for the forward (CACC) and complementary reverse (CAAA) to allow cloning at the BbSI-site of the vector plasmid. The sgRNAs sequences for *MCL-1* sgRNA1: 5′–CACCCTTATAGGTATCCACATCCG–3′; *MCL-1* sgRNA2: 5′ CACCGTCCTACAGATACCACAACC–3′. The sgRNAs sequences for *BFL-1* sgRNA1: 5′– CACCGGGAGGGCGACTTTTGGCTA–3′; *BFL-1* sgRNA2: 5′– CACCGGAGCTGGACGGGTACGAGC-3′. The sgRNA sequences were verified with U6 primers (forward primer: GAGGGCTATTTCCATGATTCC, reverse primer: GCAACACACAACATCTCCA) for successful cloning.

### Cell transfection

The *MCL-1* sgRNAs or the *BFL-1* sgRNAs were introduced into NPC HK-1 cells via transfection using Lipofectamine 2000 (Thermo Fisher Scientific). The HK-1 cells were plated at 1 × 10^5^ cells per well in 24-well plates. Three micrograms of DNA of the vector carrying either the *MCL-1* or *BFL-1* sgRNAs with 3 μl Lipofectamine 2000 were transfected into each well; cells were left for 72 h. After 72 h, transfected cells were selected with 1 ml of serum reduced media supplemented with 1 µg/ml Puromycin (Thermo Fisher Scientific). The selection lasted for 48 h. Post selection, complete media was added into each well. Once each well reached about 80% confluency, DNA and RNA were extracted from the manipulated cells to validate the gene knockouts.

### Validation of the *MCL-1 *and *BFL-1* knockouts

*MCL-1* and *BFL-1* mutagenesis were verified by conventional Sanger DNA sequencing and quantitative PCR (qPCR). The genomic knockouts of both genes were screened with customized PCR primers approximately ~ 100–200 bp within the CRISPR cut site. MCL-1 sgRNA1: Forward oligo: 5′-GTT TGG CCT CAA AAG AAA CG-3′; Reverse oligo: 5′-CTC TCT ATC CCC CTC CCC-3′. MCL-1 sgRNA2: Forward oligo: 5′-CCG CTT GAG GAG ATG GAA G-3′; Reverse oligo: 5′-TAT TGT GGT CAT GCC TGC CCG-3′. *BFL-1 sgRNA1:* Forward oligo: 5′-AGC CTC CGT TTT GCC TTA TC– 3′; Reverse oligo: 5′-GAA GGG GTC AAT TAC TAC GG-3′. BFL-1 sgRNA2: Forward oligo: 5′-TCT CAG CAC ATT GCC TCA AC-3′; Reverse oligo: 5′-TCG TTT TGC AGG TCT CAC GA-3′. During the PCR assay, *Taq* polymerase (Intron Biotechnology) was used to generate the 3’A overhangs for TA cloning. The PCR products were purified and ligated into the pGEM-T Easy Vector (Promega) and later transformed into the Rubidium Chloride competent cells. The transformation culture was plated onto LBampicillin/IPTG/X-Gal plates. Successfully cloned colonies appeared white. Colonies with correct insert size were sent for sequencing (First Base). The mutated DNAs of the colonies were compared to the parental sequences to affirm the knockouts and check for the presence of InDels. Parallel to gene sequencing, gene expression quantification was performed via qPCR. The qPCR primers were customized considering the sgRNA oligo cute sites for *MCL-1* and *BFL-1*. *GAPDH* was used as the housekeeping gene and the parental cells served as controls. Primers sequences are as follows: *MCL-1*: Forward oligo: 5′-GCG GTA ATC GGA CTC AAC CT-3′; Reverse oligo: 5′-GTA GCC AAA AGT CGC CCT CC-3′. *BFL-1:* Forward oligo: 5′-GCT GGC TCA GGA CTA TCT GC-3′; Reverse oligo: 5′-TGG ACG TTT TGC TTG GAC CT-3′. *GAPDH:* Forward oligo: 5′-GTC TCC TCT GAC TTC AAC AGC G-3′; Reverse oligo: 5′-ACC ACC CTG TTG CTG TAG CCA A-3′.

### Cytotoxicity assay

Manipulated and parental cells were either treated with BCL-2 selective inhibitor ABT-199, BCL-XL inhibitor A-1331852, and MCL-1 selective inhibitor S63845, alone and in combinations. All drugs were purchased from MedChem Express, NJ, USA. Drug sensitivity assays were conducted as described previously [16]. Average IC_**50**_values were calculated from the experimental data. In the drug response curves shown, the y-axis represents cell proliferation, with cell proliferation of the untreated controls representing 100%. The x-axis was formatted to have a base 10 logarithmic scale but the drug concentrations shown were not log-transformed before plotting the graphs. Synergistic interactions between the drug combination were analysed using the CalcuSyn software (Version 2.11, Biosoft Inc, Cambridge, UK).

### Three-dimensional spheroids

Spheroids were generated as described previously [17]. Live-dead staining was conducted as described previously [18]. Spheroids were treated with A-1331852 and S63845, alone and in combination for 72 h and over 10 days. Spheroid images were taken using the Nikon-300 inverted fluorescence microscope. For quantification of green and red signals, an outline was drawn around each spheroid in a focal Z plane which showed the maximum size. Area and mean green/red fluorescence were measured, along with adjacent background readings for control spheroids, spheroids treated with either S63845 or A-1331852, and spheroids treated with a combination of the two drugs. The total corrected green/red fluorescence (TCRF) = integrated density – (area of selected cell × mean fluorescence of background readings) [19].

## Results

### Expression of the BCL-2 anti-apoptotic proteins in the NPC cell lines

**Fig. 1:**
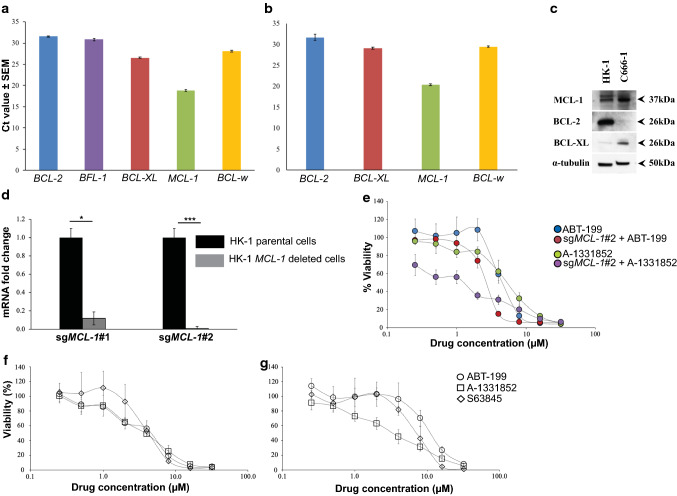
HK-1 *MCL-1* deleted cells were sensitive to treatment of BCL-XL selective inhibitor A-1331852. Expression of the anti-apoptotic genes was determined using the human apoptosis real-time PCR array (RT2 Profiler PCR Array). Ct values are inversely proportional to the amount of target gene present in the cells. The **a** HK-1 expressed all of the anti-apoptotic genes and **b** C666-1 expressed all of the anti-apoptotic genes, except *BFL-1*. Both cell lines expressed high levels of *MCL-1*. Error bars show the standard error of the mean (SEM). **c** Basal expression levels of the anti-apoptotic protein in the NPC cell lines. The basal expression levels of MCL-1, BCL-2, and BCL-XL in the C666-1 and the HK-1 cells were determined by SDS-PAGE gel electrophoresis (this image was taken from [10] with permission). **d** qPCR validation *MCL-1* gene deletion in the HK-1 cells. *MCL-1* expression levels were normalized to parental cells. Bars indicate the mean SEM of three independent experiments. Statistically significant differences in *MCL-1* expression between the parental cell line and the *MCL-1* knockout cells are shown as ***p ≤ 0.001 or *p < 0.05 determined by a two-tailed paired T-test. **e** The sensitivity of the HK-1 parental cell line and the HK-1 sg*MCL-1*#2 cells were tested to the single-agent treatment of either ABT-199 or A-1331852 (0–32 μM). Points represent the SD of four experiments. **f** HK-1 and **g** C666-1 were treated with increasing concentrations of either ABT-199, A-1331852, or S63845 (0–32 μM). Points represent the SD of four experiments

The basal expressions of all the anti-apoptotic genes in the NPC cell lines HK-1 and C666-1 were first determined using a human apoptosis real-time PCR array. In the HK-1 cells, all the anti-apoptotic genes were detected. Most of the anti-apoptotic genes required more than 20 cycles of PCR amplification to detect except for *MCL-1* which was detectable within 18 cycles of PCR amplification, which indicated a high expression level of this gene in the cells (Fig. 1a). In the C666-1 cells, all of the anti-apoptotic genes were detectable except *BFL-1*. Similar to the HK-1 cells, *MCL-1* was detected within 20 cycles in the C666-1 cells (Fig. 1b). Taken together, data shows that both NPC cell lines expressed all of the anti-apoptotic genes, except for *BFL-1* in the C666-1 cells. More notably, both NPC cell lines expressed high levels of *MCL-1*. At the protein level, both NPC cell lines expressed the anti-apoptotic protein MCL-1. A high expression level of BCL-2 was detected in the HK-1 cells, whereas a relatively high expression level of BCL-XL was detected in the C666-1 cells (Fig. 1c).

### Inhibition of either MCL-1, BCL-2, or BCL-XL alone is not sufficient to kill NPC cells

Given the high expression levels of *MCL-1* in both NPC cell lines, the functional relevance of *MCL-1* for NPC cell survival was determined by deleting the gene in the NPC cell lines, using the CRISPR/Cas9 technique. The NPC cell lines HK-1 and C666-1 were transfected with two independent single-guide RNAs (sgRNAs) targeting different regions of the human *MCL-1* gene (hereafter the sgRNAs will be referred to as sg*MCL-1*#1 and sg*MCL-1*#2). *MCL-1* mutagenesis was verified by conventional Sanger DNA sequencing and gene expression of *MCL-1* in the parental and *MCL-1* deleted cells was verified by qPCR. The HK-1 sg*MCL-1*#2 cells demonstrated a higher number of InDels compared to the HK-1 sg*MCL-1*#1 (Additional file 1: Fig. S1). The HK-1 sg*MCL-1*#2 cells demonstrated complete reduction of *MCL-1* compared to HK-1 sg*MCL-1*#1 cells which only resulted in a reduction of *MCL-1* expression by 90% (Fig. 1d). The HK-1 sg*MCL-1*#2 cells were viable despite a complete reduction of MCL-1 indicating that the cells did not rely on MCL-1 alone for survival. The C666-1 sg*MCL-1*#2 cells demonstrated a higher number of InDels compared to the C666-1 sg*MCL-1*#1 cells (Additional file 2: Fig. S2a). The C666-1 sg*MCL-1*#1 and sg*MCL-1*#2 cells only resulted in a reduction in *MCL-1* expression by 77% and 80%, respectively (Additional file 2: Fig. S2b).

Given that the sg*MCL-1*#2 demonstrated a more profound reduction of *MCL-1* expression, the HK-1/C666-1 sg*MCL-1*#2 cells were treated with increasing concentrations of either ABT-199 or A-1331852 to access whether *MCL-1* deletion sensitized NPC cells to these inhibitors. The HK-1 parental cells were resistant to single-agent treatment of ABT-199 and A-1331852 (Fig. 1e). The HK-1 sg*MCL-1*#2 cells were weakly sensitized to ABT-199 (Fig. 1e—red circle and Additional file 3: Table S1). However, the HK-1 sg*MCL-1*#2 cells were sensitized to A-1331852 by ~ fourfold (Fig. 1e—purple circle and Additional file 3: Table S1), indicating that the MCL-1 and BCL-XL may be important for NPC cell survival. The C666-1 sg*MCL-1*#2 cells were not sensitive to single-agent ABT-199 and single-agent A-1331852 (Additional file 2: Fig. S2c), most probably due to incomplete reduction of *MCL-1*.

To complement the gene-editing study, the individual contribution of MCL-1, BCL-2, and BCL-XL for NPC cell survival was parsed using BH3 mimetics which selectively inhibit these proteins. The NPC cell lines HK-1 and C666-1 were treated with either single-agent ABT-199, A-1331852, or S63845. The HK-1 (Fig. 1f) and C666-1 (Fig. 1g) cells were resistant to single-agent treatment of all three BH3-mimetics suggesting that the cells depend on more than one anti-apoptotic protein for survival.

### NPC cell lines were sensitive to co-inhibition of MCL-1 and BCL-2

**Fig. 2:**
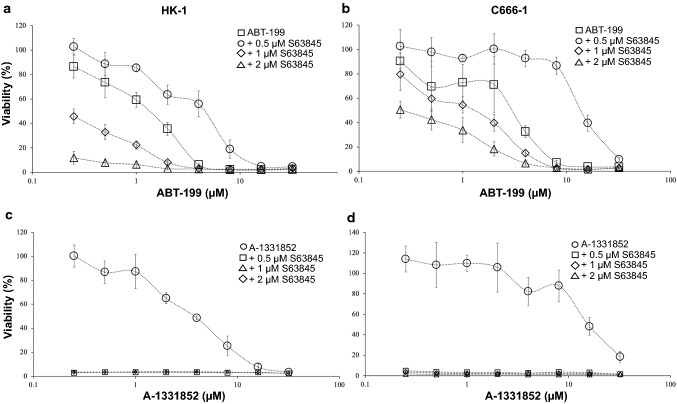
Co-inhibition of BCL-2 and MCL-1 using ABT-199 and S63845. **a** HK-1 cells were treated with increasing concentrations of ABT-199 (0–32 μM) in the presence and absence of S63845. Points represent the SD of four experiments. **b** C666-1 cells were treated with increasing concentrations of ABT-199 (0–32 μM) in the presence and absence of S63845. Points represent the SD of four experiments. Co-inhibition of BCL-XL and MCL-1 using A-1331852 and S63845. **c** HK-1 and **d** C666-1 cells were treated with increasing concentrations of A-1331852 (0–32 μM) in the presence and absence of S63845. Points represent the SD of four experiments

Given that the NPC cells were insensitive to the single-agent treatment of the BH3 mimetics, the cells were first tested combination of ABT-199 and S63845. The HK-1 cells were treated with increasing concentrations of ABT-199 (0–32 µM) in the absence and presence of either 0.5, 1, or 2 µM of S63845 for 72 h. At 0.5 µM S63845, the cells were only sensitized to ABT-199 by threefold (Fig. 2a—open circle and Additional file 4: Table S2). The sensitization increased to 16-fold at a concentration of 1 µM (Fig. 2a—open triangle and Additional file 4: Table S2) and 2 µM of S63845 (Fig. 2a—open diamond and Additional file 4: Table S2). Drug interaction analyses were conducted using the CalcuSyn software (Version 2.11, Biosoft Inc, Cambridge, UK). Drug interaction analyses indicated that the drug combinations demonstrated synergism at multiple doses of combinations of ABT-199 and S63845 in the HK-1 cells (Additional file 5: Table S3).

Similarly, the C666-1 cells were treated with increasing concentrations of ABT-199 (0–32 µM) in the absence and presence of either 0.5, 1, or 2 µM of S63845 for 72 h. At 0.5 µM S63845, the cells were sensitized to ABT-199 by fivefold (Fig. 2b—open circle and Additional file 4: Table S2). The sensitization increased to 12-fold at 1 µM (Fig. 2b—open triangle and Additional file 4: Table S2) and increased further to 36-fold at 2 µM of S63845 (Fig. 2b – open diamond and Additional file 4: Table S2). Drug interaction analyses indicated that the drug combinations demonstrated synergism at multiple doses of combinations of ABT-199 and S63845 in the C666-1 cells (Additional file 6: Table S4).

### Substantial inhibition of NPC cell proliferation driven by co-inhibition of MCL-1 and BCL-XL

Next, the NPC cell lines were treated with increasing doses of A-1331852 (0–32 µM) and fixed doses of S63845 (0.5 µM, 1 µM or 2 µM) for 72 h. In the HK-1 cells, the presence of 0.5 µM S63845, complete 100% cell killing was achieved resulting in 0% cell viability (Fig. 2c—open square) and S63845 sensitized the cells to A-1331852 by > 15-fold (Additional file 7: Table S5). Similar data were obtained when the concentration of S63845 was increased to 1 µM (Fig. 2c—open diamond and Additional file 7: Table S5) and 2 µM (Fig. 2c—open triangle and Additional file 7: Table S5).

Similarly, in the C666-1 cells, combination with S63845 sensitized the cells to A-1331852 to all three concentrations tested. In the presence of 0.5 µM S63845, there was a complete loss of the dose-dependent curve resulting in 0% cell viability (Fig. 2d—open circle) and S63845 sensitized the cells to A-1331852 by > 29-fold (Additional file 7: Table S5). Similar data were obtained when the concentration of S63845 was increased to 1 µM (Fig. 2d—open diamond and Additional file 7: Table S5) and 2 µM (Fig. 2d—open triangle and Additional file 7: Table S5).

### S63845 sensitized spheroids to A-1331852 and vice versa

Next, a combination of S63845 and A-1331852 was tested on spheroids generated from the HK-1 cells. The HK-1 spheroids were treated with S63845 and A-1331852 alone and in combination for 3 days.

**Fig. 3:**
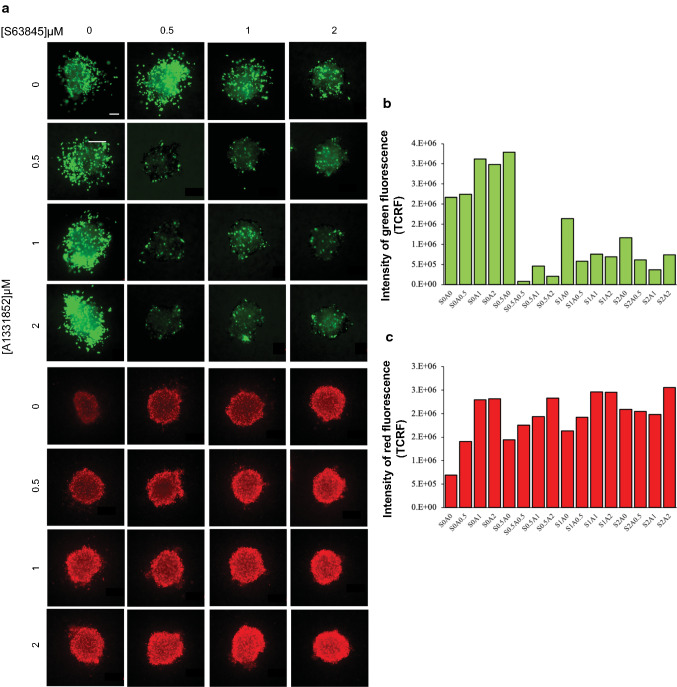
The effect of the combination of S63845 and A-1331852 on the growth and invasion of HK-1 spheroids over 3 days. **a** The spheroids were treated with single agents S63845 and A-1331852 and a combination of both over three days at the indicated concentrations, n = 2–3 spheroids per combination. Cell viability was determined using the live/dead assay (Viable cells: stained green by Calcein-AM; Dead cells: stained red by Ethidium-homodimer I). Size bar: 200 μm. The intensity of **b** green and **c** red fluorescence was measured for each drug combination and presented as TCRF, *n* = 2 spheroids per combination. TCRF: Total Corrected Red Fluorescence; “S” denotes S63845 and “A” denotes A-1331852. The numbers next to “S” and “A” indicate the doses of the drug combinations

Spheroids were insensitive to the single-agent treatment of S63845 and A-1331852 except at 2 µM concentration of each drug (Fig. 3a). There was obvious sensitization of the spheroids to A-1331852 by S63845 at a concentration of S63845 as low as 0.5 µM. Similarly, at 0.5 µM of A-1331852, the spheroids were sensitized to S63845 (Fig. 3a). Further increase in concentrations of both inhibitors, reduced the viability of the spheroids in a dose-dependent manner (Fig. 3a). This was evident with the decrease in green fluorescence intensity which indicates spheroid viability (Fig. 3b) but an increase in red fluorescence intensity which indicates cell death (Fig. 3c), indicating the presence of more dead cells as the combination concentrations increased.

Collectively, our data demonstrated that there was a greater response of the NPC cell lines to co-inhibition of MCL-1 and BCL-XL compared to co-inhibition of MCL-1 and BCL-2, suggesting that co-inhibition of MCL-1 and BCL-XL are better therapeutic targets for killing NPC cells.

### *BFL-1* deleted NPC cells sensitized to BCL-XL selective inhibitor A-1331852

**Fig. 4:**
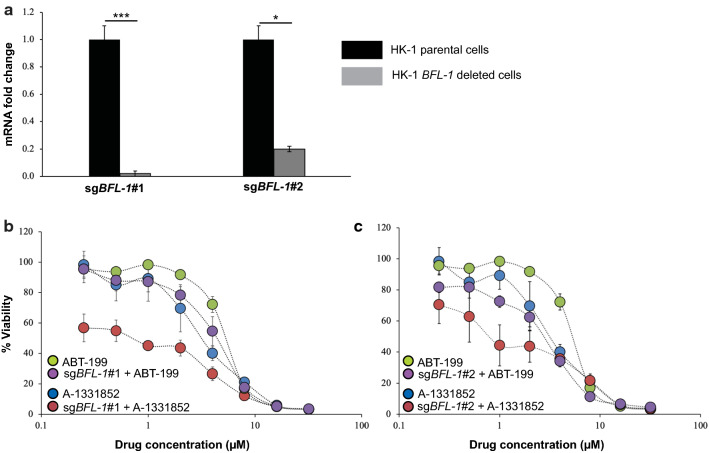
HK-1 *BFL-1* deleted cells were sensitive to treatment of BCL-XL selective inhibitor A-1331852. **a** Quantitative PCR (qPCR) validation *BFL-1* gene deletion in the HK-1 cells. *BFL-1* expression levels were normalized to parental cells. Bars indicate the SD of three independent experiments. Statistically significant differences in *BFL-1* expression between the parental cell line and the *BFL-1* knockout cells are shown as ***p ≤ 0.001 or *p < 0.05 determined by a two-tailed paired T-test. The sensitivity of the HK-1 parental cell line and the **b** HK-1 sg*BFL-1*#1 and **c** HK-1 sg*BFL-1*#2 cells were tested to single-agent treatment of either ABT-199 or A-1331852 (0–32 μM). Points represent the SD of four experiments

The C666-1 cells expressed undetectable levels of *BFL-1* (Fig. 1b). Hence, to access the role of *BFL-1* in cell survival, the gene was deleted in the HK1 cells. The HK-1 cells were transfected with two independent single-guide RNAs (sgRNAs) targeting different regions of the human *BFL-1* gene (hereafter the sgRNAs will be referred to as sg*BFL-1*#1 and sg*BFL-1*#2). *BFL-1* mutagenesis was verified by conventional Sanger DNA sequencing and gene expression of *BFL-1* in the parental and *BFL-1* deleted cells were verified by qPCR. The sg*BFL-1*#1 and sg*BFL-1*#2 resulted in a reduction in *BFL-1* expression by ~ 98% and 80%, respectively (Fig. 4a).

The sg*BFL-1*#1 and sg*BFL-1*#2 cells were treated with increasing concentrations of either ABT-199 or A1331852 to access whether *BFL-1* deletion sensitized NPC cells to these inhibitors. The HK-1 parental cells were resistant to single-agent treatment of ABT-199 and A-1331852 (Fig. 4b, c). The sg*BFL-1*#1 (Fig. 4b and Additional file 8: Table S6) and the sg*BFL-1*#2 (Fig. 4c and Additional file 8: Table S6) cells were weakly sensitized to ABT-199. However, the sg*BFL-1*#1 (Fig. 4b and Additional file 8: Table S6) and the sg*BFL-1*#2 (Fig. 4c and Additional file 8: Table S6) cells were sensitized to A-1331852 by ~ fivefold and fourfold, respectively indicating that the BFL-1 and BCL-XL may be important for NPC cell survival.

## Discussion

Given that different cell population is addicted to different anti-apoptotic protein(s) for survival, it is crucial to determine the anti-apoptotic proteins that NPC cells depend on for survival. We combined the CRISPR/Cas9 technique and BH3 mimetics to delineate the individual contributions of the anti-apoptotic proteins for NPC survival. BH3 mimetics, given their highly selective inhibition of the anti-apoptotic proteins, provide a chemical toolkit to parse the individual contributions of the anti-apoptotic proteins for cancer cell survival.

Complete deletion of *MCL-1* alone did not kill the HK-1 sg*MCL-1*#2 cells, implying that other anti-apoptotic proteins may be compensating for the loss of *MCL-1*. Similarly, the NPC parental cell lines were insensitive to the single-agent treatment of S63845. Moreover, the NPC parental cell lines were also insensitive to the single-agent treatment of ABT-199 and A-1331852 indicating that NPC cells depend on more than one anti-apoptotic protein for survival. The HK-1 sg*MCL-1*#2 cells were weakly sensitized to ABT-199 but were sensitized to A-1331852 indicating that MCL-1 and BCL-XL may be important for NPC cell survival. Although the HK-1 sg*MCL-1*#2 cells were sensitized to A-1331852, the effect of the sensitization was weaker compared to the effect observed when the BH3-mimetics were used. The effect of co-inhibition MCL-1 and BCL-XL with S63845 and A-1331852 were more profound as both the HK-1 and C666-1 cell lines were instantly killed at nanomolar drug combination concentrations. The difference in effect between the genetic knockout of *MCL-1* and its pharmacological effect could be attributed to the use of bulk *MCL-1* knockout cells for the drug sensitivity assays. The population of cells used was heterogeneous, thus, inhibition of *MCL-1* may not be as phenotypically severe compared to pharmacological inhibition of MCL-1. The inter-clonal heterogeneity might have a strong influence on cell survival and hence it is challenging to determine the complete effects of *MCL-1* inhibition, in the NPC cells. The HK-1 and C666-1 cells also responded to co-inhibition of MCL-1 and BCL-2 but synergism was mostly attained at higher concentrations of the drugs (> 1 µM) demonstrating that inhibition of MCL-1 and BCL-XL are better therapeutic targets for killing NPC cells.

Given the promising sensitivity of the NPC cells to co-inhibition of BCL-XL and MCL-1 in monolayer culture, co-inhibition of BCL-XL and MCL-1 was tested in NPC spheroids. The sensitization obtained in the monolayer culture was similar to findings obtained with the spheroid studies. Spheroids generated from the HK-1 cells were sensitized to A-1331852 by S63845 and vice versa, indicating that the combination may be effective in vivo. Similar to our findings, several recent studies demonstrated that solid tumours depend on BCL-XL and MCL-1 for survival and pharmacological inhibition of these proteins resulted in cell killing. For example, co-targeting MCL-1 and BCL-XL resulted in reduced cell viability of cells isolated from squamous cell carcinoma of the head and neck (SCCHN), in in vitro and in vivo models [20]. Combination targeting MCL-1 and BCL-XL synergistically killed melanoma cell lines in vitro [21]. Paediatric solid tumour cell lines, namely rhabdomyosarcoma, Ewing sarcoma, osteosarcoma, and neuroblastoma cell lines were co-dependant on BCL-XL and MCL-1 for survival. Co-treatment with A-1331852 and S63845 induced rapid cell killing of these cell lines in vitro and in vivo models [22]. Our study demonstrated that co-inhibition of MCL-1 and BCL-XL was crucial for killing cervical cancer cell lines [16, 17].

One possible mechanism that may have led to the profound loss of cell viability of the NPC cells, as a consequence of co-inhibition of MCL-1 and BCL-XL, could be explained by the relationship between BIM, MCL-1, and BCL-XL. MCL-1 and BCL-XL sequester BIM and inhibition of either MCL-1 or BCL-XL liberate BIM which can be later sequestered by the uninhibited protein [23–25]. We speculate that co-targeting MCL-1 and BCL-XL in the NPC cell lines may have led to complete freedom of BIM which in turn led to activation of BAX and BAK which resulted in the activation of cell death in the NPC cells.

Although a majority of solid tumours depend on BCL-XL and MCL-1 for survival, targeting BCL-XL and MCL-1 in the clinic may be cumbersome for multiple reasons. MCL-1 and BCL-XL are crucial for the survival of not only tumour cells, but also for non-malignant cells. Hence, targeting these molecules is toxic to normal cells. For example, inhibition of BCL-XL results in thrombocytopenia [26, 27], and inhibition of MCL-1 was reported to result in cardiac toxicity [28, 29]. Co-inhibition of BCL-XL and MCL-1 was previously reported to result in fatal hepatotoxicity [30]. There are a few strategies to overcome this issue. Our findings show that cell killing can be achieved at very low doses (nanomolar ranges) of S63845 and A-1331852, which may not be sufficient to cause thrombocytopenia or cardiac toxicity. A recent finding demonstrated that co-inhibition of MCL-1 and BCL-XL was feasible in a zebrafish model of head and neck squamous cell carcinoma [20] and embryonic chicken model of rhabdomyosarcoma (RMS) [22]. Another strategy would be to co-inhibit BCL-2 and MCL-1. Although the effect of inhibiting BCL-2 and MCL-1 was not as pronounced as inhibiting BCL-XL and MCL-1, a combination of ABT-199 and S63845 synergistically inhibited cell proliferation of the NPC cell lines. Hence, targeting BCL-2 and MCL-1 could be an alternative treatment strategy. Although still preliminary, co-targeting BCL-XL and BFL-1 could be an alternate strategy to reduce toxicities associated with co-inhibiting MCL-1 and BCL-XL. Targeting BFL-1 in addition to BCL-XL should not add significant toxicity to cells, as loss of *BFL-1* was reported to only reduce certain T-cell subpopulations and dendritic cells [31]. BFL-1 by large is not essential for the development and survival of most normal and healthy tissues [31] compared to MCL-1 and BCL-XL which are crucial for the survival of some non-malignant cells. Hence, targeting BFL-1 and BCL-XL may result in an acceptable therapeutic window without causing much damage to the normal cells.

There was no notable reduction in the cell viability observed in the BFL-1 manipulated cells despite the complete decrease in *BFL-1* expression level, especially in the HK-1 sg*BFL-1*#1 cells. The sg*BFL-1#1* cells were equally viable as the sg*BFL-1#2* cells which did not display complete *BFL-1* reduction, suggesting that the *BFL-1* alone is not essential for NPC cell survival, highlighting the need for additional inhibition of other anti-apoptotic proteins in this context. Treatment with A-1331852 and not ABT-199, sensitized both sg*BFL-1*#1 *and* sg*BFL-1*#2 cells to the drug, indicating that BFL-1 and BCL-XL may be necessary for NPC cell survival. The role of BFL-1 in cancer cell survival is not consistent when different cell populations are considered. For example, RNAi-mediated silencing of *BFL-1* was not sufficient to induce cell death in lymphoma cell lines. However, pharmacological inhibition of MCL-1 with AZD5991, in combination with dose-dependent *BFL-1* knockdown, induced cell killing, demonstrating that BFL-1 and MCL-1 were crucial for the survival of the lymphoma cells. The same study reported that the *BFL-1* knockdown in lymphoma cells was insensitive to pharmacological inhibition of BCL-XL and BCL-2 [32]. In a separate study, CRISPR/Cas9 mediated knockout of *BFL-1* in melanoma cells were not sensitized to pharmacological inhibition of MCL-1, BCL-XL, and BCL-2 indicating that the melanoma cells do not rely on BFL-1 for survival [21].

The importance of BCL-XL and BFL-1 for NPC cell survival still requires further interrogation. The contribution of *BFL-1* for NPC cell survival was only interrogated in one NPC cell line, hence our findings may be a cell-type dependant effect. The functional importance of *BFL-1* should be interrogated in additional NPC cell lines to omit the possibility of cell-type dependant effect. Furthermore, inhibition of *BFL-1* via CRISPR might not be phenotypically severe compared to the immediate inhibition of BFL-1 with a BH3-mimetic. CRISPR-modified cell lines are subjected to evolutionary pressure over days or weeks. The intense pressure of Cas9-induced modifications may select for secondary mutations that blunt any anti-proliferative consequences of the original mutation [33]. However, the unavailability of BFL-1 selective BH3-mimetic halts any further investigation on the role of BFL-1 in promoting the survival of NPC cells.

Taken together, our findings reiterate the importance of determining the anti-apoptotic proteins that cancer cells depend on for survival so that the cells can be targeted optimally to achieve maximal cell killing. In the context of NPC, MCL-1 and BCL-XL are crucial for NPC cell survival and co-targeting these proteins kill cells at low drug doses. Although preliminary, interrogation on the role of *BFL-1* for NPC cell survival provided new insight on the potential of BFL-1 as a therapeutic target for NPC treatment.

## Supplementary Information

**Additional file 1.** Read sequences of the HK-1 sg*MCL-1*#1 and HK-1 sg*MCL-1*#2 cells. Parental MCL-1 sequence is shown on the top. Clones tested showed deletions at the expected cleavage sites (arrow). The dashed lines indicate the InDels. The target sequence is highlighted in yellow while the PAM sequence is highlighted in red.

**Additional file 2.** (**a**) Read sequences of the C666-1 sg*MCL-1*#1 and C666-1 sg*MCL-1*#2 cells. Parental MCL-1 sequence is shown on the top. Most of the clones tested showed deletions at the expected cleavage sites (arrow). The dashed lines indicate the InDels. The target sequence is highlighted in yellow while the PAM sequence is highlighted in red. (**b**) qPCR validation *MCL-1* gene deletion in the C666-1 cells. *MCL-1* expression levels were normalized to parental cells. Bars indicate mean SEM of three independent experiments. Statistically significant differences in *MCL-1* expression between the parental cell line and the *MCL-1* knockout cells are shown as *p < 0.05 determined by two-tailed paired T-test. (**c**) The sensitivity of the C666-1 parental cell line and the HK-1 sg*MCL-1*#2 cells were tested to tested to single agent activity of either ABT-199 or A-1331852 (0-32 μM). Points represent SD of four experiments.

**Additional file 3.** Sensitivity of the HK-1 NPC cell line to either ABT-199 or A-1331852 following manipulation of *MCL-1*.

**Additional file 4.** Sensitization of NPC cell lines to ABT-199 by S63845 (fold sensitization).

**Additional file 5.** The synergistic drug effects of ABT-199 and S63845 in the HK-1 cell. The combination index values were calculated using the CompuSyn software. [ ] indicates drug concentration; CI shows combination index values, CI<1 indicates synergism, CI=1 indicates additive and CI >1 indicates antagonism.

**Additional file 6.** The synergistic drug effects of ABT-199 and S63845 in the C666-1 cell. The combination index values were calculated using the CompuSyn software. [ ] indicates drug concentration; CI shows combination index values, CI<1 indicates synergism, CI=1 indicates additive and CI >1 indicates antagonism.

**Additional file 7.** Sensitization of NPC cell lines to A-1331852 by S63845 (fold sensitization).

**Additional file 8.** Sensitivity of the HK-1 NPC cell line to either ABT-199 or A-1331852 following manipulation of *BFL-1*.

## Data Availability

All data are available from the corresponding author upon reasonable request.
