# Supplementary material for: Dual inhibition of anti-apoptotic proteins BCL-XL and MCL-1 enhances cytotoxicity of Nasopharyngeal carcinoma cells
Source: Discov Oncol. 2022 Feb 3;13:9. doi: 10.1007/s12672-022-00470-9 (PMC8814124; doi:10.1007/s12672-022-00470-9)
Supplement: Supplementary file 3 — Additional file 3. Sensitivity of the HK-1 NPC cell line to either ABT-199 or A-1331852 following manipulation of MCL-1. [file 12672_2022_470_MOESM3_ESM.docx]

**Supplementary Table 1:** Sensitivity of the HK-1 NPC cell line to either ABT-199 or A-1331852 following manipulation of *MCL-1*.

| **Drug** | **Cell Type** | **IC_50_ ± SD (µM)** | **Fold sensitization** |
| --- | --- | --- | --- |
| ABT-199 | Parental HK-1 cell line | 4.58 ± 0.44 |  |
|  | HK-1 sg*MCL-1*#2 cells | 2.64 ± 0.12 | 1.7 |
| A-1331852 | Parental HK-1 cell line | 5.18 ± 1.22 |  |
|  | HK-1 sg*MCL-1*#2 cells | 1.18 ± 0.15 | 4.4 |

NOTE: Fold sensitization was computed relative to the parent cell line, as shown.
