# Supplementary material for: Dual inhibition of anti-apoptotic proteins BCL-XL and MCL-1 enhances cytotoxicity of Nasopharyngeal carcinoma cells
Source: Discov Oncol. 2022 Feb 3;13:9. doi: 10.1007/s12672-022-00470-9 (PMC8814124; doi:10.1007/s12672-022-00470-9)
Supplement: Supplementary file 4 — Additional file 4. Sensitization of NPC cell lines to ABT-199 by S63845 (fold sensitization). [file 12672_2022_470_MOESM4_ESM.docx]

**Supplementary Table 2:** Sensitization of NPC cell lines to ABT-199 by S63845 (fold sensitization).

| **Cell line** | *S63845 (µM)* | **ABT-199**  IC_50_ ± SD (µM) | **Fold sensitization by S63845** |
| --- | --- | --- | --- |
| **HK-1** | *0* | 4.2 ± 1.3^a^ | - |
|  | *0.5* | **1.3 ± 0.2^b^** | 3.2** |
|  | *1* | **<0.25^b^** | 16.8*** |
|  | *2* | **<0.25^b^** | 16.8*** |
| **C666-1** | *0* | 13.8 ± 1.25^a^ | - |
|  | *0.5* | **2.7 ± 0.72^b^** | 5.1**** |
|  | *1* | **1.2 ± 0.51^b^** | 11.6**** |
|  | *2* | **0.38 ± 0.23^b^** | 36.3**** |

NOTE: The IC_50_ values are concentrations of **drug 1** (bold) that killed 50% of the cells surviving the shown concentrations of *drug 2* (italics). Fold sensitization: IC_50a_ / IC_50b_. Where the parent IC_50_ was not calculable, the lower bound was used. Statistically significant differences with the IC_50_ of ‘a’ are shown as **p ≤ 0.01 or ***p ≤ 0.001 or **** p ≤ 0.0001 determined by two-tailed paired T test.
