# Supplementary material for: Dual inhibition of anti-apoptotic proteins BCL-XL and MCL-1 enhances cytotoxicity of Nasopharyngeal carcinoma cells
Source: Discov Oncol. 2022 Feb 3;13:9. doi: 10.1007/s12672-022-00470-9 (PMC8814124; doi:10.1007/s12672-022-00470-9)
Supplement: Supplementary file 5 — Additional file 5. The synergistic drug effects of ABT-199 and S63845 in the HK-1 cell. The combination index values were calculated using the CompuSyn software. [ ] indicates drug concentration; CI shows combination index values, CI<1 indicates synergism, CI=1 indicates additive and CI >1 indicates antagonism. [file 12672_2022_470_MOESM5_ESM.docx]

**Supplementary Table 3: The synergistic drug effects of ABT-199 and S63845 in the HK-1 cell.** The combination index values were calculated using the CompuSyn software. [ ] indicates drug concentration; CI shows combination index values, CI<1 indicates synergism, CI=1 indicates additive and CI >1 indicates antagonism.

| ABT-199 [µM] | S63845 [µM] | CI |
| --- | --- | --- |
| 0.25 | **0.5** | 1.15 |
| 0.5 |  | 0.678 |
| 1 |  | 0.548 |
| 2 |  | 0.359 |
| 4 |  | 0.09 |

| ABT-199 [µM] | S63845 [µM] | CI |
| --- | --- | --- |
| 0.25 | **1** | 0.29 |
| 0.5 |  | 0.202 |
| 1 |  | 0.159 |
| 2 |  | 0.074 |
| 4 |  | 0.046 |

| ABT-199 [µM] | S63845 [µM] | CI |
| --- | --- | --- |
| 0.25 | **2** | 0.08 |
| 0.5 |  | 0.059 |
| 1 |  | 0.06 |
| 2 |  | 0.035 |

Antagonism

Synergism
