# Supplementary material for: Dual inhibition of anti-apoptotic proteins BCL-XL and MCL-1 enhances cytotoxicity of Nasopharyngeal carcinoma cells
Source: Discov Oncol. 2022 Feb 3;13:9. doi: 10.1007/s12672-022-00470-9 (PMC8814124; doi:10.1007/s12672-022-00470-9)
Supplement: Supplementary file 6 — Additional file 6. The synergistic drug effects of ABT-199 and S63845 in the C666-1 cell. The combination index values were calculated using the CompuSyn software. [ ] indicates drug concentration; CI shows combination index values, CI<1 indicates synergism, CI=1 indicates additive and CI >1 indicates antagonism. [file 12672_2022_470_MOESM6_ESM.docx]

**Supplementary Table 4: The synergistic drug effects of ABT-199 and S63845 in the C666-1 cell.** The combination index values were calculated using the CompuSyn software. [ ] indicates drug concentration; CI shows combination index values, CI<1 indicates synergism, CI=1 indicates additive and CI >1 indicates antagonism.

| ABT-199 [µM] | S63845 [µM] | CI |
| --- | --- | --- |
| 0.25 | **0.5** | 0.338 |
| 0.5 |  | 0.146 |
| 1 |  | 0.248 |
| 2 |  | 0.387 |
| 4 |  | 0.192 |
| 8 |  | 0.085 |

| ABT-199 [µM] | S63845 [µM] | CI |
| --- | --- | --- |
| 0.25 | **1** | 0.279 |
| 0.5 |  | 0.155 |
| 1 |  | 0.175 |
| 2 |  | 0.165 |
| 4 |  | 0.097 |
| 8 |  | 0.041 |

| ABT-199 [µM] | S63845 [µM] | CI |
| --- | --- | --- |
| 0.25 | **2** | 0.175 |
| 0.5 |  | 0.150 |
| 1 |  | 0.134 |
| 2 |  | 0.094 |
| 4 |  | 0.058 |
| 8 |  | 0.049 |

Synergism
