# Supplementary material for: Dual inhibition of anti-apoptotic proteins BCL-XL and MCL-1 enhances cytotoxicity of Nasopharyngeal carcinoma cells
Source: Discov Oncol. 2022 Feb 3;13:9. doi: 10.1007/s12672-022-00470-9 (PMC8814124; doi:10.1007/s12672-022-00470-9)
Supplement: Supplementary file 8 — Additional file 8. Sensitivity of the HK-1 NPC cell line to either ABT-199 or A-1331852 following manipulation of BFL-1. [file 12672_2022_470_MOESM8_ESM.docx]

**Supplementary Table 6:** Sensitivity of the HK-1 NPC cell line to either ABT-199 or A-1331852 following manipulation of *BFL-1*.

| **Drug** | **Cell Type** | **IC_50_ ± SD (µM)** | **Fold sensitization** |
| --- | --- | --- | --- |
| ABT-199 | Parental HK-1 cell line | 5.28 ± 0.23 | - |
|  | HK-1 sg*BFL-1*#1 cells | 4.29 ± 0.71 | 1.2 |
|  | HK-1 sg*BFL-1*#2 cells | 2.68 ± 0.24 | 2 |
| A-1331852 | Parental HK-1 cell line | 3.06 ± 0.46 | - |
|  | HK-1 sg*BFL-1*#1 cells | 0.67 ± 0.24 | 4.5 |
|  | HK-1 sg*BFL-1*#2 cells | 0.81 ± 0.12 | 4 |

NOTE: Sensitization was computed relative to the parent cell line, as shown.
